# Microwave induced thermally assisted solvent-based bonding of biodegradable thermoplastics: an eco-friendly rapid approach for fabrication of microfluidic devices and analyte detection

**DOI:** 10.1038/s41598-022-20257-w

**Published:** 2022-09-27

**Authors:** Md Sadique Hasan, Shayan Borhani, Sai Sathish Ramamurthy, Abhay Andar, Xudong Ge, Fow-Sen Choa, Yordan Kostov, Govind Rao

**Affiliations:** 1 Center for Advanced Sensor Technology, University of Maryland Baltimore County, 1000 Hilltop Circle, Baltimore, MA 21250 USA; 2 Department of Computer Science and Electrical Engineering, University of Maryland Baltimore County, 1000 Hilltop Circle, Baltimore, MA 21250 USA; 3 Department of Chemical, Biochemical and Environmental Engineering, University of Maryland Baltimore County, 1000 Hilltop Circle, Baltimore, MA 21250 USA; 4 STAR Laboratory, Department of Chemistry, Sri Sathya Sai Institute of Higher Learning, Prasanthi Nilayam, Puttaparthi, Anantapur, Andhra Pradesh 515134 India; 5 Potomac Photonics Inc., Process and Product Technologies, 1450 South Rolling Road, Baltimore, MA 21227 USA

**Keywords:** Lab-on-a-chip, Techniques and instrumentation

## Abstract

There is an increasing interest in low-cost, facile and versatile thermoplastic bonding for microfluidic applications that can be easily transitioned from laboratory prototyping to industrial manufacturing. In addition, owing to the surge in the usage of thermoplastic microfluidics and its adverse effect on the environment, it is prudent to source alternative materials that are biodegradable, providing a sustainable, green approach. To address the problems, here we introduce an environment friendly, low-cost and safe welding technology used in the fabrication of microcassettes from biodegradable cellulose acetate (CA) thermoplastics. The thermally assisted solvent based bonding of the thermoplastics was accomplished in a domestic microwave oven with the aid of a polyether ether ketone (PEEK) vise. To characterize the quality of the bonding, our in-house technique was compared with a conventional thermally assisted solvent bonding configuration using a heat press machine and tested under different conditions. Our microwave induced bonding of CA presents three times reduced bonding time with higher bonding strength, good reliability and does not necessitate the use of cumbersome instrumentation. Finally, we demonstrate an electrophoresis application and vitamin C detection accomplished using this biodegradable microcassette presenting comparable results with traditional techniques, illustrating the potential of this fabrication technique in different microfluidic applications.

## Introduction

Microfluidics has become a powerful technology in the last few decades and has found applications in several frontier research areas that include analytical chemistry^1^, pharmaceuticals^2^, synthesis of chemicals^3^ and clinical application^4,5^. Requirement of less sample and reagent volumes, high separation efficiency, high surface-to-volume ratio, rapid analysis, high degree of sensitivity, and the ability to be integrated into a lab-on-a-chip make this platform more appealing and a popular choice over the conventional methods that involve large scale equipment that is cost intensive and cumbersome. However, the requirement for complex fabrication techniques, cleanroom facilities and advanced microfabrication equipment hinder the path toward mass production of microfluidic systems. Thermoplastic materials in this regard have achieved a significant breakthrough as an alternative to conventional materials i.e., glass and polydimethylsiloxane (PDMS)^6^, for a variety of applications. This includes the use of materials such as polymethyl methacrylate (PMMA)^7^, cyclic olefn copolymer (COC)^8^ or polycarbonate (PC)^9^.

Bonding thermoplastics is a frequent process often encountered in the fabrication of microfluidic devices. Conventional methods for bonding microfluidic chips to one another can be accomplished using several bonding techniques such as thermal^10–13^, UV^10,14^, solvent^10,15^, microwave^10^, and friction spot welding^16^ methodologies. Solitarily these bonding methods exhibit some disadvantages such as channel deformation, dimension loss, and the requirement of high-end instrumentation^13,17–19^ which limits the applications of these methods. Thermally assisted solvent bonding techniques yield an appropriate bonding quality and coverage^13^ providing sturdy, clean bonds and good sealing properties by taking advantage of the solvent-based methods and eliminating its limitations. This technique is based on dissolving thermoplastic in a poor solvent at elevated temperature on account of the decline in the solvents’ solubility parameter that occurs by increasing the temperature with the aid of clamping force^20,21^. By solvent evaporation, activated acrylate monomers on both of the substrates weld to one another^22,23^ and form a fully bonded device.

Microwave heating has been a popular choice for food processing, but more recently has proven beneficial for chemical synthesis^24^ and metal sintering^25^. Microwave-induced thermally assisted solvent bonding has been a green approach with the optimization of the process parameters for minimizing channel distortions and comparable bonding strengths with other bonding methods. In the few earlier reported publications on microwave based bonding^26,27^, the mechanism of heating were similar to the disposable susceptors used but it requires a custom microwave setup or hot embossers. An alternate approach is using the metal paper clips to provide the clamping force using the heat absorbed by the clips^28^. The drawback of this method is that it must be monitored during the heating process to ensure that the clips do not become overheated and melt the substrate. Further, reusing the metal clips might be amenable to a shorter melting time. Considering the rapidly achievable and tunable thermal assisted solvent based bonding without compromising bonding quality and strength, shorter fabrication time and non-essentiality of customized machines, it is of immense importance to pin down alternate means to easily and safely bond the thermoplastic substrates for different microfluidic applications.

With the advancement of microfluidic devices, the usage of thermoplastic materials is also on the rise. As most of the thermoplastics are not biodegradable, it has resulted in large scale pollution and an environmental threat. Biodegradable materials are hence a favorable choice over the persistent thermoplastic products that result in large scale environmental pollution^29,30^. As a result, a green approach to microfluidic device fabrication is an emerging field providing sustainable outcomes^31^. A good candidate with excellent optical, mechanical, and thermal properties is cellulose acetate (CA). The cellulose acetate is a biomass substance obtained by chemically modifying hydroxyl groups with acetic acid in cellulose materials obtained from wood fibers and cotton fibers etc. CA is a widely used chemically modified natural polymer whose applications vary from the textile industry to plastic films, packaging, and cigarette filter tows^32^ with the attractive properties of biodegradability^33^, hardness, good impact resistance, optical transparency, resistance to hydrocarbons and lack of static electricity^34,35^. These features make CA an ideal choice to be implemented in microfluidic applications and this study. Our previous experience comprises sustainable material based works^31,36^ and now we are extending that knowledge to CA.

In this study, we have implemented a user friendly and rapid vise-based method to weld biodegradable CA thermoplastic substrates in the microwave induced thermal assisted solvent-based bonding technique. Ethanol has been chosen to weld the thermoplastic sheets owing to its controllability, the smaller difference in solubility of CA, preservation of optical transparency and little or no deformation of the microfluidic channels. A laser cutter is used for rapid prototyping and iterative design of microfluidic devices easily in a time-efficient manner. Despite their high cost, they are comparable to CNC milling and 3D printing and do not require time lengthy techniques and cleanroom facilities for microfluidic chip fabrication. The vise material and screws are made of PEEK which has excellent chemical and mechanical properties at high temperatures and more importantly invisible to microwave irradiation^37^. The vise is used to implement the clamping force necessary to weld the two sheets which reduces the complications of any material melting inside the microwave and also does not require a cost intensive setup. To compare the performance of this green chemistry approach involving microwave assisted vise-based method to a conventional thermally assisted solvent based bonding method, a heat press machine was also used. Several tests were conducted including bonded coverage area test, burst test, transmittance test and surface characteristics to evaluate the bonding performance. To the best of our knowledge, in this study, we are demonstrating for the first time the fabrication of a biodegradable CA microcassette based on thermally assisted solvent based welding using microwave radiation. Additionally, in the supplementary information, PMMA bonded substrates were also tested in terms of the aforementioned tests, and the results showed that the CA bonded substrates had similar or better performance. Lastly, as proof of concept, a simple and quick electrophoresis study and smartphone based detection of vitamin C were conducted with the CA bonded devices utilizing the microwave assisted technique.

## Methods and materials

### Fabrications methods and equipment

The CA sheets used in this study were diacetate cellulose (DAC) (San Diego plastic, USA) having dimensions of 400 mm 550 mm of different thicknesses. As the laser micromachining offers high flexibility in the control of channel dimensions and design based on the ability to control the laser parameters^38^, smaller substrates were directly patterned using the laser cutter. Microfluidic channels were designed in CorelDRAW Graphic Suite X4 and converted to laser paths with the laser cutter built-in software. The CA sheets were cut into substrates according to the requirements using a VersaLaser 3.60 CO$_2$ laser cutter having a power rating of 60 W. The power of the laser was set constant to 100% while the speed was varied to engrave or vector cut the CA substrates. Prior to bonding or analysis, engraved CA sheets were cleansed with ethanol and subsequently with dry clean wipes. A change in coloration to yellow was noticeable in the laser cut region of CA and was catered to by rinsing with ethanol that resolved the coloration.

**Figure 1:**
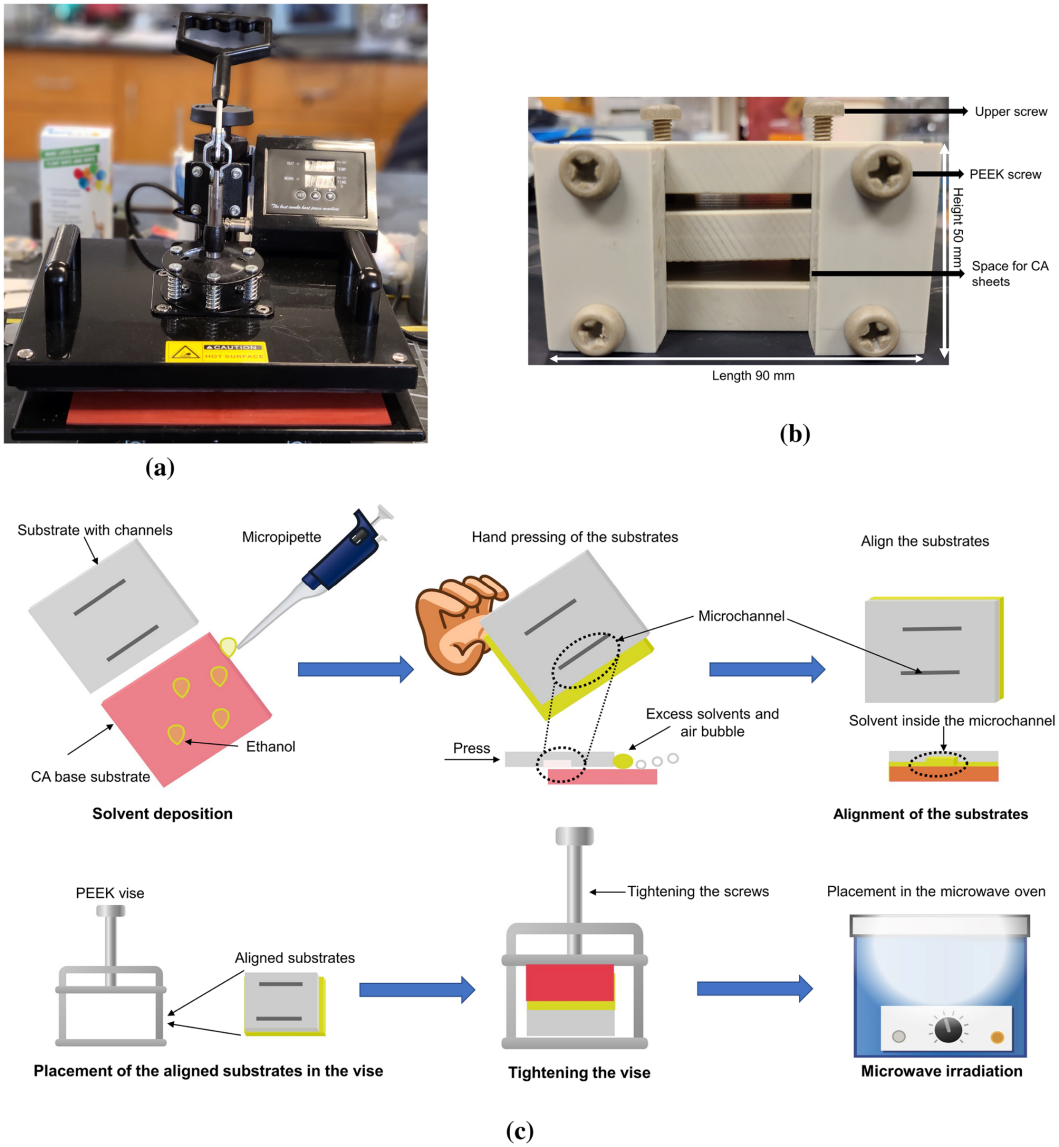
(**a**) Heat press used for thermally assisted bonding. (**b**) Photograph and specifications of laboratory cast PEEK vise used for microwave bonding. (**c**) Schematic illustration of the overall procedure performed for CA bonding with the microwave.

The heat press machine presented in Fig. 1a (SUNCOO 16x20IN Heat Transfer Machine) was used in this study, as it has the capability of controlling the temperature of the silicone rubber platen and the time required for bonding. The temperature of the heat press machine was set to 80 $^{\circ }$C for all of the experiments conducted in this study. Different iterations and time durations were attempted using high, medium and low pressure settings according to the datasheet^39^ of the heat press machine to optimize the bonding parameters. A silicone sheet cut to the dimension of the CA sheet was placed on the silicone rubber platen to serve as a control for alignment and to avoid surface damage while heating and pressing.

The microwave heating system used in this study consisted of a domestic microwave oven (Emerson MW8987W) which had a power rating of 1.35 kW and an operating frequency of 2.45 GHz. The microwave was operated for different time scales to optimize the parameters at 100% power for CA bonding.

The PEEK based vise cast in our lab has a dimension of 90 mm × 50 mm × 50 mm. The PEEK material (Mcmaster-Carr, USA) had a thickness of 0.5 in. or 12.7 mm sheets, drilled and cut according to the need of the vise. The screws were also purchased from the same company. A picture of our PEEK based vise is shown in Fig. 1b. As can be seen, the upper two screws can be rotated clockwise or counterclockwise to either tighten or loosen the sheets placed inside, before and after bonding. The two screws were tightened to the same extent alternatively to create a balanced force on the microfluidic device placed inside the vise.

Absolute ethanol has been solely used as the solvent for bonding. This technique is based on dissolving CA in ethanol at an elevated temperature decreasing the solvents’ solubility parameter^20,21^. Ethanol being a weak solvent in CA, the surface wetting treatment only swells the first few microns of the material which is fully reversible after thermally induced evaporation of the solvent. This method is more reliable and controllable than other solvents such as acetone, chloroform which dissolve CA in ambient temperature, has the potential drawback to clog or distort the channel dimensions. Ethanol was taken in a syringe or micropipette and sprayed on the surface of the sheet to cover the whole surface of the CA to be bonded without flooding (typically 100 $\upmu$l for 50 mm × 20 mm surface). The CA sheets to be bonded are then handpressed for 10 s ensuring no air bubbles are formed inside and then inserted into the vise and the screws were rotated clockwise to tighten the hold and the whole setup was placed in the microwave with the entire procedure taking less than a minute. The full procedure for the bonding via microwave is shown in Fig. 1c.

### Performed tests and procedure

**Figure 2:**
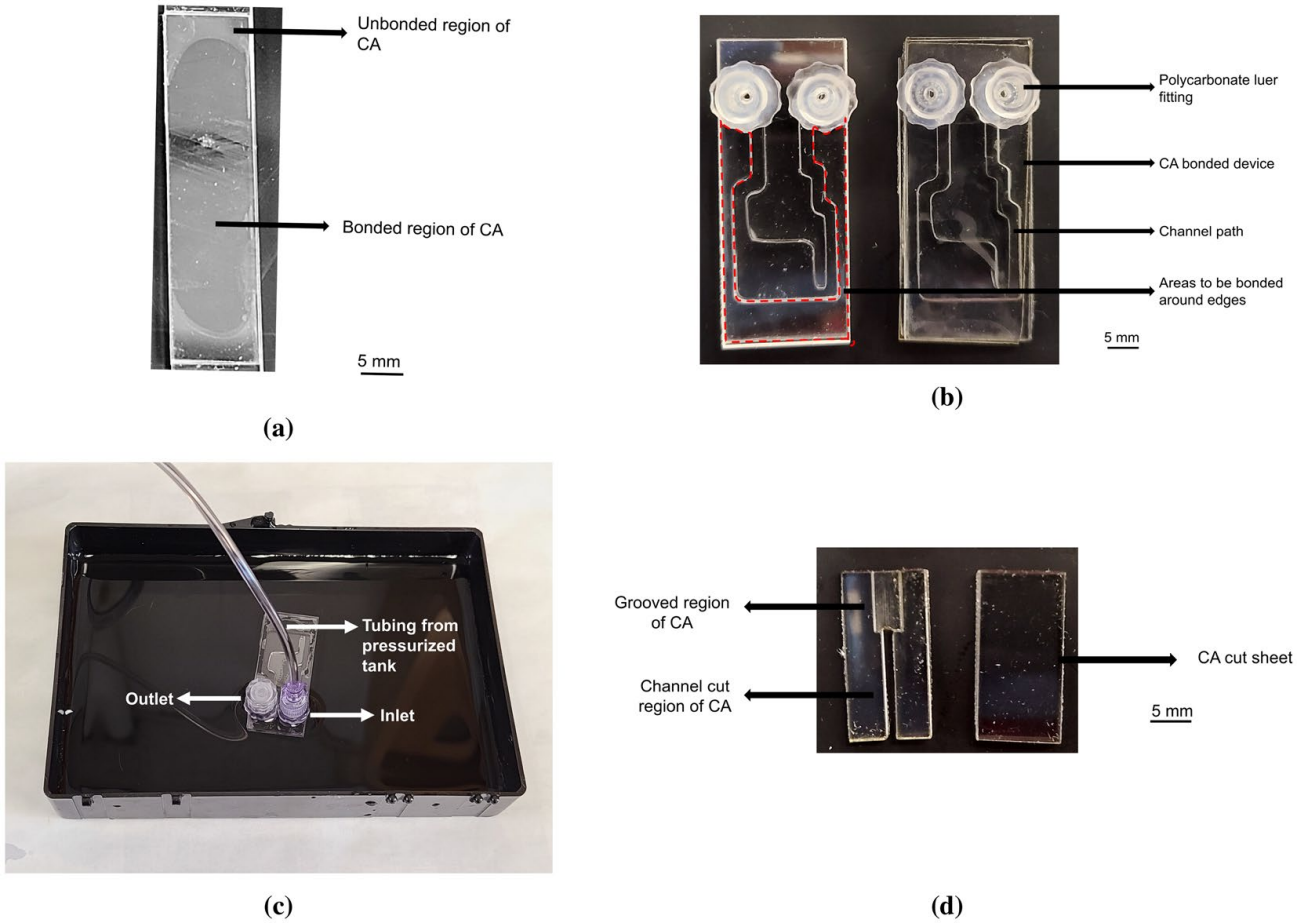
(**a**) Photograph of CA bonded device with unbonded and bonded regions. Bonded regions are darker than the unbonded regions. (**b**) Photograph of CA bonded device with relevant components for leakage and burst tests. (**c**) CA bonded device in operation during leakage or burst test with inlet operating from a pressurized tank using tygon tubing and outlet is locked with luer lock fitting. (**d**) Photograph of laser cut CA with channel cut and grooved regions for surface characteristic test of the sheets to be bonded.

To quantify the optimized process parameters and compare the performance of different solvent bonding procedures, several tests have been performed. The first and primary test implemented was the bonded coverage area test. The CA sheets of thickness 1.5 mm were cut in 25 mm × 25 mm dimensions and were bonded. The two different surfaces involved in the bonding were examined both visually and under a microscope for bonding coverage^40,41^ (Fig. 2a) and then using the ImageJ software^42^ to determine the percentage of bonded area for every configuration. Generally, the bonded regions were darker than the unbonded regions. Based on the initial bonded area test using the heat press machine and microwave induced bonding, the better configurations for CA were continued for further testing.

The leakage and burst tests were performed as presented in the device architecture (Fig. 2b) with two 1.5 mm thick CA sheets and one 0.2 mm thick CA sheet as a cover sheet. Two important features of this design are to be noted; small areas to be bonded around the edges and a very thin layer as a protection layer; were implemented to explore the reliability, vulnerability and bonding strength of different methods apart from conventional leakage and burst tests’ design. These features were expected to play an important role in assessing the device bonding strength and susceptibility in different microfluidic applications. For the leakage and burst tests, the inlet was connected to a pressurized airflow that can go up to 60 psi and the outlet was locked. The inlet and outlet were connected and blocked using the polycarbonate luer lock fittings. The device was placed under water (Fig. 2c) and the flow was increased until air bubbles were seen in the water coming from the bonded device. Same configurations were applied to evaluate the leakage test as well, only the pressure was slightly increased from zero and the cassette was inspected for air bubbles originating from the CA bonded devices. A statistically significant value was obtained by performing the leakage and burst tests seven times each.

The transmittance of the bonded and separate CA sheets (laid on top of one another) was performed using a spectrophotometer (Spectramax M5). Each of the sheets was 1.5 mm thick and the transmittance was calculated from the optical density provided by the instrument from 250 to 800 nm for different bonding configurations.

The surface and the channel properties before and after bonding were inspected both visually and under an AmScope microscope^43^. To verify the effect of bonding, a vector cut channel of width 1.5 mm and a grooved region of thickness approximately 0.75 mm out of 1.5 mm of length 10 mm was patterned using the laser cutter. A sample image with the laser cut CA sheets is shown in Fig. 2d. The channel dimension, the grooving and the intersection between the vector cut channel and the rasterized area were inspected pre- and post-bonding and any deformations on the regions were noted. The widths and deformations were evaluated using ImageJ software^44^ and the thickness of the grooved region was measured using a slide caliper.

### Proof of concept

As a proof of concept of the bonded CA devices, electrophoresis, a method to separate molecules according to factors such as size, shape and charge by applying an electric field has been performed utilizing the microwave used in bonding studies. A very facile, low-cost electrophoresis operation has been performed with microwave induced CA bonded device. The full experimental details of the electrophoresis system in a custom CA framework with simple pencil leads are described in the supplementary information.

Vitamin C alternatively known as ascorbic acid is a water soluble dietary nutrient that is vital in a variety of biological functions^45^. There are various approaches for the determination of vitamin C of which chromatographic technique and capillary electrophoresis are the most commonly used^46^. In this context, resazurin dye (7-hydroxy-3H-phenoxazin-3-one10-oxide) has been widely used as a redox indicator of cell viability in several types of proliferation and cytotoxicity assays^47,48^. Resazurin is a weakly fluorescent entity (absorption peak at 600 nm), however, in the presence of a reducing reagent, a pink color evolves due to the formation of the highly fluorescent resorufin (fluorescent peak 570 nm)^49^. In this study, ascorbic acid (vitamin C) has been used to reduce resazurin to resorufin, which resulted in a turn on fluorescence signal. The increase in fluorescence intensity is directly related to the extent of resazurin reduction which in turn is directly proportional to the quantity of vitamin C added to the assay solution. The fluorescence intensity was quantified using a smartphone camera to determine the level of vitamin C addition. An elaborate procedure for this proof of concept study is included in the supplementary information section.

## Results and discussions

### Post bonding results and analysis of conducted tests

To quantify the optimized process parameters and compare the performance of different solvent bonding procedures, several tests have been performed using the heat press machine and microwave after bonding the CA substrates. Different time durations were attempted using both the machines and different pressure settings were initiated in the case of the heat press machine.

#### Bonded area test

**Figure 3:**
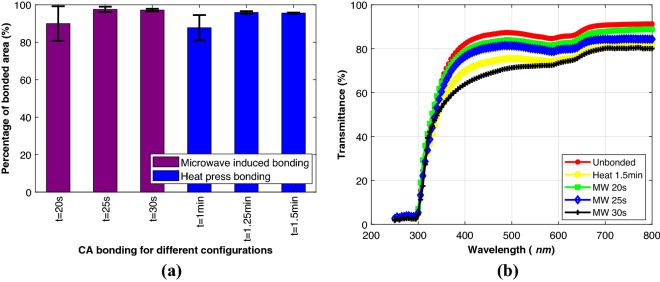
(**a**) The average bonded area (%) for different configurations of CA bonding using ImageJ software. The X-axis denotes different configurations and Y-axis denotes the percentage of bonded area. Minute is represented by “min” and second by “s”. (**b**) The transmittance (%) after bonding for different CA configurations. The ‘MW’ represents microwave induced and ‘Heat’ represents heat press based bonding in the figure. Minute is represented by “min” and second by “s”.

The heat press machine was operated in high, medium, and low-pressure configurations for varying time scales. It was observed that at low pressure configuration and for less than 1 min ethanol did not completely evaporate to aid with the bonding successfully. Further, for the high-pressure configuration, the channels appeared to be broken or heavily damaged. So, in the case of the heat press machine, for CA bonding 1.25- and 1.5-min time duration were carried out using the medium pressure setting. The higher limit on the time duration was chosen such that exceeding this time would damage the channel and surface of the bonding, confirmed by visual inspection of the thermoplastics. For the microwave induced bonding, different time durations were tried upon setting the microwave power to 100%. The same criteria as the heat press machine for the lower and higher limit of time duration for bonding were applied. For CA bonding 20, 25- and 30-s time duration were used. The complete list of different configurations is presented in Fig. 3a for CA along with their bonded area percentage evaluated as mentioned previously. Each of the experiments was performed twice and the average is shown in Fig. 3.

From the results, for the heat press-based bonding, the maximum bonded coverage area configuration is 1.25 min. For the microwave induced bonding, the maximum bonded coverage area configuration is 25 s. For both cases, the maximum bonded coverage areas for CA are nearly equal. But it was observed from the results that, the microwave induced bonding takes significantly less time than conventional heat press-based bonding. This is on account of improved softening and interpenetration of the polymers accomplished upon microwave irradiation, which is more efficient in the presence of ethanol. A similar experiment was performed with PMMA substrates and the results are illustrated in the supplementary Fig. S1. The results show a comparable bonded area percentage with less bonding time required for CA. For the heat press-based bonding, 1 min CA bonding was discontinued from the later experiments as it yielded the lowest percentage of bonded areas.

#### Leakage and burst test

The next set of tests performed were the leakage and burst tests. These two tests were performed under similar conditions in duplicates and were designed to explore the bonding strength, vulnerability and reliability of the bonding method. No further burst tests were conducted for the configurations where the leakage was found (Table 1). The maximum bonding strength was calculated based on the average of the seven burst tests’ pressure performed. It is observed from the burst test data, the maximum bonding strength configuration is 1.5 min for the heat press bonding. For the microwave induced bonding, the time taken to achieve maximum bonding strength is 30 s. Hence, the microwave induced bonding takes significantly less time to successfully bond than the other heat press-based bonding. In addition to that, the maximum bonding strength is significantly much higher for microwave induced bonding. In terms of the vulnerability and reproducibility of the bonded device, it can be seen that the microwave induced bonding delivers a more successfully bonded device than the heat press-based bonding. 5 out of 14 (35%) devices were observed to leak while 3 out of 21 (14%) devices was found faulty in case of microwave induced bonding. The reason might be in case of the microwave-assisted solvent bonding, the organic solvent is not only used for bonding the CA substrates but also serves as an energy receptor to absorb the microwave irradiation. It was verified that the heating of the CA substrate without the solvent is negligible. It should be noted that ethanol presents a high dielectric constant ($\epsilon _s$ = 24.5) to absorb microwave energy at the bonding interface. As a result of the dielectric heating mechanism, the organic solvent absorbs microwave energy and selectively heats at the bonding interface. By accelerating organic solvent diffusion by microwave irradiation, physical polymer chain interlocking is achieved, resulting in stronger bonds and a more successful bonding process. This is in contrast to the conventional thermal bonding approach where the heat is transmitted from the polymer substrate to the solvent layer to bond the device. This process usually requires a long time and is less efficient since polymers are materials with low thermal conductivity. The leakage and burst tests were also performed with the PMMA and result is shown in supplementary Table 1. The microwave induced bonded CA performed superiorly to PMMA in the tests. In terms of bonding strength, the 30 s for CA are the best configurations that are significantly higher than the heat press based bonding.

**Table 1:** Average burst pressures along with percent defective device for different configurations of CA bonding via the heat press and microwave techniques. Seven iterations have been performed for each of the configurations.

Material	Heat press configuration	Average burst pressure (psi)	Percent defective (%)	Microwave configuration	Average burst pressure (psi)	Percent defective (%)
CA	1.25 min	2.1	57.1	20 s	10.4	42.9
	1.5 min	8.7	14.3	25 s	36.7	0
				30 s	56.9	0

#### Transmittance test

Transmittance is an important criterion in different microfluidics applications, especially where optical clarity is required. From the leakage and burst test performance, the 1.25 min for CA were discontinued due to low reliability and as the study is primarily focused on microwave assisted solvent bonding. The transmittance test was performed with blank and bonded 1.5 mm thick sheets and the percentage of transmittance was compared (Fig. 3b). From Fig. 3b, the blank CA has the highest transmittance in the visible region. With the increase in microwave time for bonding, the transmittance drops. From, 20 to 25 s the transmittance drops 3% and 5% respectively from blank while for other configurations it drops approximately 10% for heat press-based bonding and 20% for microwave based 30 s bonding. With more microwave time, the layers are more heated via ethanol resulting in better fusion, but this damages the glassy finish of the sheets with haze. The experimental result with PMMA is shown in supplementary Fig. S2 and the same trend with increasing time was observed.

#### Surface characteristics test

**Figure 4:**
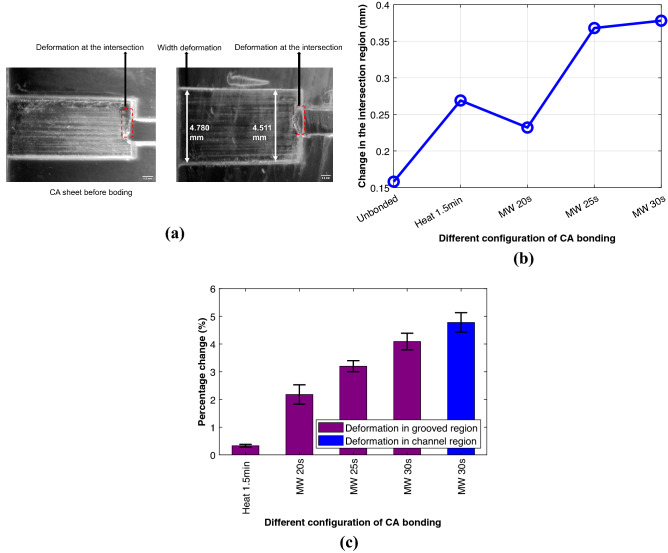
(**a**) (Left) Microscopic photographs of laser cut CA sheets before bonding. The CA sheet has a slight deformation at the intersection of grooved and channel cut region. (Right) Post bonding CA sheet for 30 s microwave bonding. (**b**) Change in the intersection region in mm for different configurations of CA bonding. (**c**) Percentage change in the grooved and channel cut region for different configurations of CA bonding. The ‘MW’ represents microwave induced and ‘Heat’ represents heat press based bonding. Minute is represented by “min” and second by “s”.

The surface and channel characteristics were also inspected pre- and post-bonding for different bonding configurations. The CA sheets were cut in such a way so that the channel and groove properties can be explored after bonding. The dimensions, thickness of the channel and grooved region were inspected with the microscope and compared with the blank. Further, the change in the width of the grooved region and the channel region was noted. A slight deformation on the blank CA is observed as presented in Fig. 4a at the intersection of the grooved region and vector cut channel region. This is likely due to the laser heat stress initiated by the abrupt change of temperature during laser cutting at the intersection. The deformation in this intersected region increased with increasing time in the microwave induced bonding (Fig. 4b). This can be ascribed to the solvent stress created by the vise ethanol combination, higher solvent concentration and longer microwave time. Due to the solvent stressing, the grooved region width was noticed to behave similarly as evidenced in Fig. 4c. This small increase ($\sim$ 2 to 4%) in the grooved region width is prominent on the far side of the channel region as illustrated in Fig. 4a. No distortion was observed in the channel cut regions except for 30 s bonding of CA in the vicinity of the rasterized region. Hence, deformation was initiated and amplified by the solvent stress, laser cutting and longer heating time.

#### Discussion

It can be seen from the different tests’ results that there is no single bonding configuration that is superlative in all of the tests. The exact bonding configuration can be chosen based on the design suited for the intended application of the bonded device. But based on the test parameter results, the overall best bonded configuration can be accredited to the microwave assisted 25 s for CA. This bonding yields higher bonding strength, nearly equal transmittance to the blank and does not show deformation in the vector cut region. Although this configuration provides less bonding strength than 30 s bonding, it has better transmittance and less deformation in addition to the lesser time required for the device fabrication.

Hence, our microwave assisted thermal based solvent bonding offers multi-dimensional advantages being the ease of fabrication, green approach, lesser fabrication time required and no requirement of cumbersome instrumentation for bonding of thermoplastics. For CA it has reduced from 1.5 min to 30 s when compared to the conventional thermally assisted solvent based bonding. There is an overall two and a half to three times reduction in bonding time without the requirement of sophisticated machines to bond. This paves the way for the microfluidic application considering reduced time, biodegradability, good bonding strength and more reliable bonding.

### Proof of concept electrophoresis study

**Figure 5:**
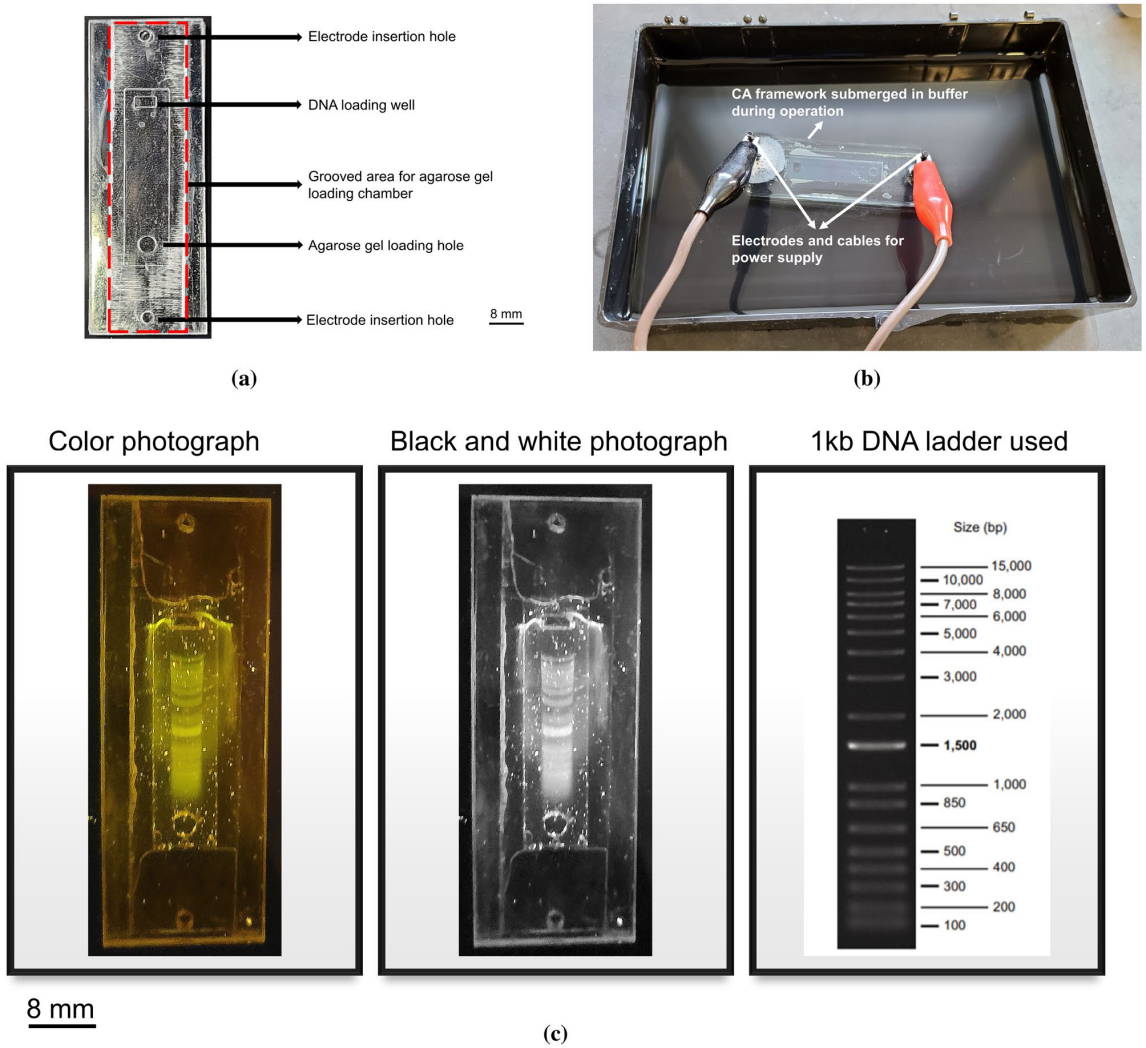
(**a**) Photograph of bonded CA framework for electrophoresis operation. (**b**) Electrophoresis setup in operation with bonded CA device with pencil lead electrodes and cables. (**c**) (Left two photographs) Black and white and color photograph after electrophoresis operation using 1 Kb DNA via an e-gel safe imager. (Right) Standard 1 Kb DNA ladder. Image from: https://www.goldbio.com/product/14/1-kb-dna-ladder.

Different configurations for facilitating a simple, low-cost electrophoresis technique have been attempted. The CA framework for loading the agarose gel, DNA and electrodes has been prototyped and bonded in a similar manner using the ideal parameters of the microwave induced solvent assisted bonding (Fig. 5a). For the application of the electric field through the gel, a normal Cu wire was glued to the electrode position via colloidal silver paste and the power supply was provided. For the electrophoresis operation, the operating voltage was 50 V in each initiation providing an electric field intensity of 8 V/cm in our current microcassette framework. The current measured by the power supply module was 3–4 mA in this case. Pencil lead served as an alternative means to Cu wire as electrodes (Fig. 5b). With the pencil leads as electrodes, the current rating improved to 13–14 mA in the same configuration providing three times more current than before. This exemplifies the ability of pencil leads as efficient electrodes in the custom electrophoresis operation. After one hour of operation, Fig. 5c shows the separation by our technique using a CA bonded framework. When compared to the conventional electrophoresis results, our framework and technique can separate the 1 kb DNA as efficiently. The PMMA framework performed similarly. The frameworks can be customized for parallel and 2D electrophoresis operations as well.

**Figure 6:**
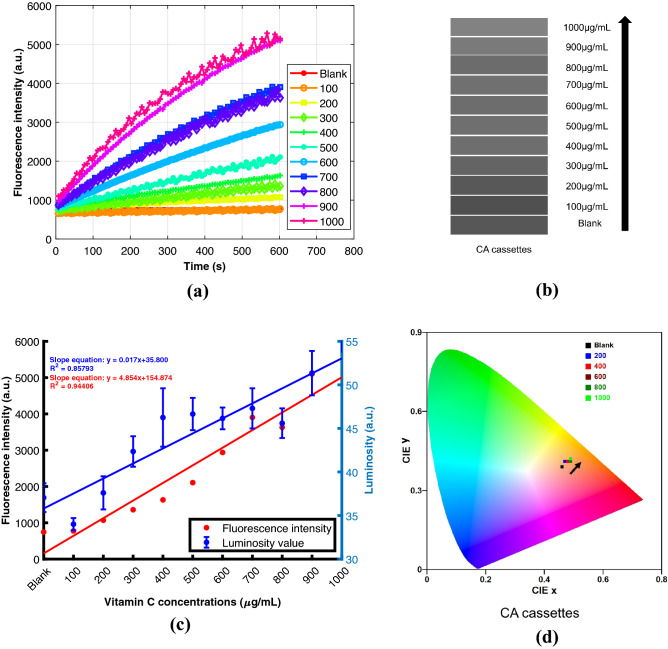
(**a**) Fluorescence intensity profile of different concentrations of vitamin C with resazurin using a spectrophotometer. The legends have the unit $\upmu$g/mL. (**b**) Gray-scale shade cards corresponding to the luminosity values for different concentrations of vitamin C for CA cassettes (**c)**) Overlap of fluorescence intensities (left y-axis) obtained from a spectrophotometer and the luminosity values (right y-axis) extracted from the mobile phone-based detector for CA. (**d**) CIE chromaticity diagram for different concentrations of vitamin C in CA cassettes.

### Proof of concept vitamin C detection

The reduction of resazurin to resorufin was monitored for fluorescence modulation for a 10 min duration in the spectrophotometer with an excitation wavelength of 530 nm and emission wavelength of 580 nm. The fluorescence spectra for different concentrations of vitamin C are presented in Fig. 6a. A consistent increase in the fluorescence intensity is observed for higher concentrations of vitamin C. To confirm the reduction to resorufin, the absorbance spectra were recorded after 10 min of addition of the resazurin, presented in Fig. S3 in the supplementary information. A gradual shift in the spectra from 600 nm to around 570 nm is observed with increasing vitamin C concentrations which confirms the reduction to resorufin. The CA cassette images along with PMMA cassettes for different concentrations inside the safe imager are presented in Fig. S4 in the supplementary information. A visible hypsochromic shift from the fluorescence is observed from different concentrations of vitamin C. The luminosity and chromaticity values were obtained from each chamber of the cassettes. The corresponding gray-scale shade card values obtained from luminosity values for the cassettes are presented in Fig. 6b. The fluorescence intensity obtained after 10 min and luminosity values processed from the android app are plotted sidewise in Fig. 6c. Although the coefficient of determination ($R^2$) of luminosity is lesser than the fluorescence intensity profile, the results from the smartphone camera show a strong correlation with increasing vitamin C concentrations. The linear regression parameters obtained from PMMA and CA cassettes are identical as illustrated in the supplementary Fig. S5. The modulation in the visible color of emission from fluorescence is also presented in the CIE chromaticity diagram in Fig. 6d and the same trend is observed as before. Due to the spectral overlap in the chromaticity diagram, only five concentrations have been plotted for both the cassettes. Therefore, our bonded devices provide a simple, fast, reliable platform to easily comprehend the spectral changes associated with any analyte of interest that can be further implemented in a point of care (POC) setting specially catering to the bottom of the pyramid.

## Conclusion

Thermoplastics substrates are emerging as an integral part of microfluidics application owing to their low cost, availability, industrial scalability, but with the possibility of negative impact after accumulating in the environment. Hence, it is of paramount importance to source alternative benign thermoplastics capable of implementation in microfluidic applications in commercial and health sectors. In this study, we have presented an easy, low-cost and green methodology for bonding biodegradable CA thermoplastics using an ordinary domestic microwave oven. We have shown reduced bonding time with comparable bonding strength of our bonded devices with other properties investigated with a conventional thermally assisted solvent based bonding. Our bonded device exhibited promising results with comparable post bonding performance. Our future study will explore bonding performance varying microwave power, different solvents, solvent concentrations and solvent deposition techniques. The facile, rapid and low-cost method, with our proof of concept microfluidic devices, could present next-gen devices that are of frugal, highly flexible, environment friendly and for use in diverse microfluidics applications.

## Supplementary Information

Supplementary Information.

## Data Availability

All data generated or analysed during this study are included in this published article and its supplementary information files.
