# Supplementary material for: Microwave induced thermally assisted solvent-based bonding of biodegradable thermoplastics: an eco-friendly rapid approach for fabrication of microfluidic devices and analyte detection
Source: Sci Rep. 2022 Sep 27;12:16075. doi: 10.1038/s41598-022-20257-w (PMC9515109; doi:10.1038/s41598-022-20257-w)
Supplement: Supplementary file 1 — Supplementary Information. [file 41598_2022_20257_MOESM1_ESM.docx]

**Supporting information - Microwave induced thermally assisted solvent-based bonding of biodegradable thermoplastics: An eco-friendly rapid approach for fabrication of microfluidic devices and analyte detection**

Md Sadique Hasan^1,2^, Shayan Borhani,^1,3^, Sai Sathish Ramamurthy^1,3,4^, Abhay Andar^1,5^, Xudong Ge^1,3^, Fow-Sen Choa^2^, Yordan Kostov^1^ and Govind Rao^1,3^.*

*^1^ Center for Advanced Sensor Technology, University of Maryland Baltimore County, 1000 Hilltop Circle, Baltimore, Maryland 21250, USA*

*^2^ Department of Computer Science and Electrical Engineering, University of Maryland Baltimore County, 1000 Hilltop Circle, Baltimore, Maryland 21250, USA*

*^3^ Department of Chemical, Biochemical and Environmental Engineering, University of Maryland Baltimore County, 1000 Hilltop Circle, Baltimore, Maryland 21250, USA*

*^4^ STAR Laboratory, Department of Chemistry, Sri Sathya Sai Institute of Higher Learning, Prasanthi Nilayam, Puttaparthi, Anantapur, Andhra Pradesh 515134, India.*

*^5^ Potomac Photonics Inc., Process and Product Technologies, 1450 South Rolling Road Baltimore, Maryland, 21227, USA*

**Table of Contents**

# Supplementary Section. Methods and Materials

Subsection. Fabrication and bonding method of PMMA

Subsection. Proof of concept

**Figure S1.** The average bonded area (%) evaluated for different configurations of PMMA bonding

**Figure S2.** The transmittance (%) calculated after bonding for different PMMA configurations

**Figure S3.** Absorbance spectrum of different concentrations of vitamin C after adding resazurin

**Figure S4**. Photograph of the PMMA and CA cassettes for different concentrations inside the safe imager

**Figure S5.** Plot of fluorescence intensities and the luminosity values for PMMA

**Table S1.** Leakage and burst test results for different configurations of PMMA bonding via the heat press and microwave techniques

**References**

**Methods and Materials:**

**Fabrication and bonding method of PMMA:**

The fabrication and bonding method of PMMA was similar to the CA as described in the method section. The PMMA sheets were cut into substrates according to the requirements using the same

CO_2_ laser cutter. The power of the laser was set constant to 100% while the speed was varied to engrave or vector cut the PMMA substrates. Before bonding or analysis, engraved PMMA sheets were cleansed with ethanol and subsequently with dry clean wipes. Ethanol was taken in a syringe or micropipette and sprayed on the surface of the sheet to cover the whole surface of the PMMA to be bonded without flooding just as CA. The PMMA sheets to be bonded are then handpressed for 10 seconds ensuring no air bubbles are formed inside and then inserted into the vise and the whole setup was placed in the microwave. Both microwave and heat press was used to evaluate the performance parameters of the bonded PMMA microfluidic devices. The leakage, burst test, transmittance tests and proof-of-concept experiments were performed for the PMMA devices as well. The fabrication and design of the microfluidic devices were also the same as the CA. Only the microwave irradiation time was changed as the time required to bond different thermoplastics will be different depending on their solubility parameters and material property.

**Proof of concept:**

For the proof of concept electrophoresis study, SYBR stained 1 kilobase (kb) DNA and ultrapure agarose powder (Invitrogen Inc, USA) were used. Agarose gel was prepared using 1X TAE buffer (50X TAE buffer diluted to 1X) and agarose powder at 1 wt %/v in the buffer, was used for studying larger molecules such as DNA base pairs from the size of 50 base pairs (bp) to thousands of bases. ^1^ The solution was then microwaved for 1.5 minutes and after every 30 seconds, it was taken out of the microwave and shaken until a clear solution appeared. The solution was then injected into the chamber and allowed to solidify. A horizontal system was used in our technique because of its simplicity and availability of premade gels and buffers.^2^ Based on recent studies, pencil graphite electrodes (PGE) can be used directly or embedded in insulating polymers. Commercial pencil leads are finding applications in polarography,^3^ electrodes and diffracting objects in electrochemical studies^4^ and as working electrodes for voltammetry.^5^ Simple pencil leads (0.2 mm, 2B) have been used as electrodes for the electrophoresis technique used in this study. A Copper (Cu) wire attached with colloidal silver paste has also been tried but the current rating is much improved with the use of the pencil lead-based electrophoresis technique. A custom CA microcassette framework were designed and bonded using microwave assisted technique to perform the electrophoresis experiment. The DNA solution was placed in a well inside the gel with pencil electrode insertion points. During the injection of agarose gel solution, the well region for loading DNA was masked using a CA cut substrate and taken out after solidification providing a space for DNA solution deposition. The power supply was provided from an electrophoresis power supply module (BioRad PowerPac, USA). The whole system was then submerged in the buffer to facilitate electric field application through the gel for DNA separation. After the electrophoresis operation, the imaging of the separation of DNA was done by an e-gel safe imager (Invitrogen Inc, USA).

For the proof of concept vitamin C detection study, ten different concentrations (wt%/v) of vitamin C solution were prepared in 1X Phosphate-buffered saline (PBS) buffer ranging from 100 *µ*g/mL (568 *µ*M) to 1000 *µ*g/mL (5.68 mM) in regular intervals with a blank ascorbic acid powder in this case (USP/EP). The range was carefully chosen so that resazurin is reduced to resorufin and does not convert further to colorless dihydro resorufin. 5 *µ*M resazurin was added in each solution and the fluorescence intensity was measured in a spectrophotometer for 10 minutes to monitor the reduction of resazurin with time. After 10 minutes, the solution was transferred into the CA cassettes as shown in Figure [2a](#_bookmark1). The cassettes were placed in an Invitrogen G6600 safe imager 2.0 and the images of each cassette chamber were captured using a cell phone camera (Samsung galaxy S20). The images were later processed in the “Color Grab”6 android application to analyze the luminosity and chromaticity for different concentrations of vitamin C with the blank. Duplicate experiments were performed and average values were taken for analysis.

**Supplementary figures**

The average bonded area (%) evaluated for different configurations of PMMA bonding is shown in Fig. S1. The percentage of bonded areas are comparable to the CA. Further, one notable observation is that the CA sheets require significantly less bonding time than the PMMA, which can be attributed to the difference in their polymeric structure and solubility in the solvent used.


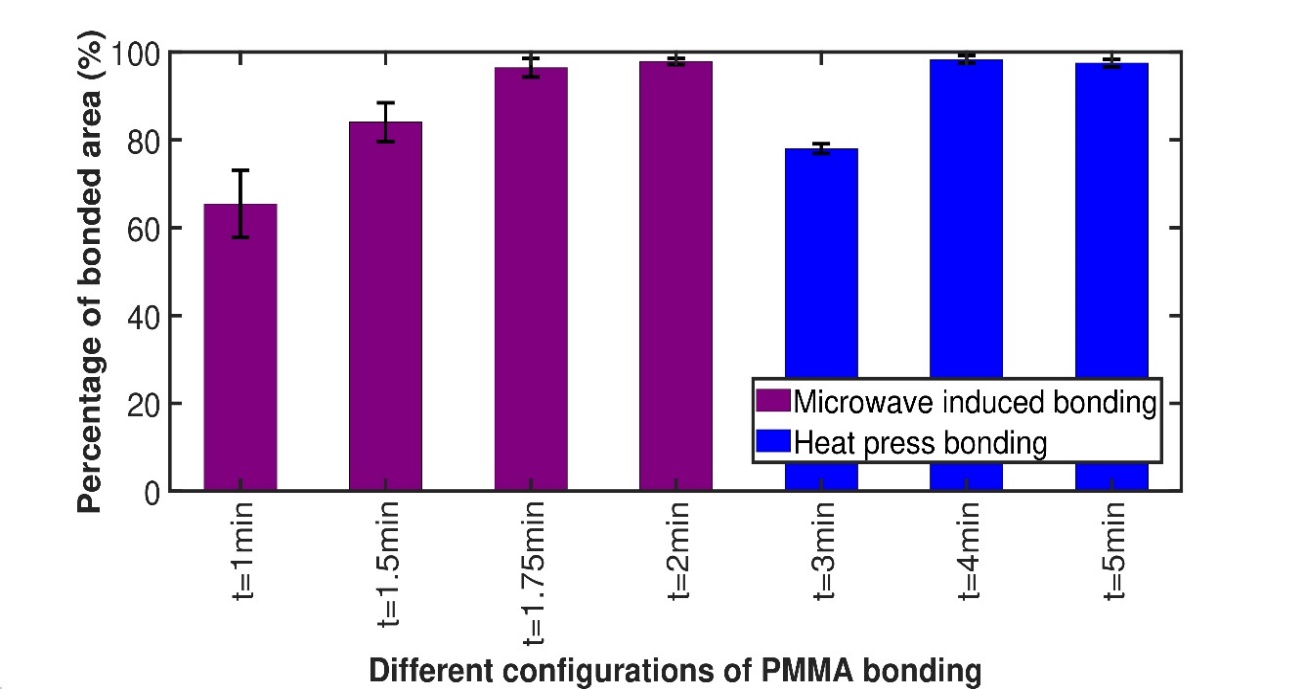


**Fig.** **S1.**  The average bonded area (%) for different configurations of PMMA bonding using ImageJ software. The X-axis denotes different configurations and Y-axis denotes the percentage of bonded area. Minute is represented by "min" and second by "s".


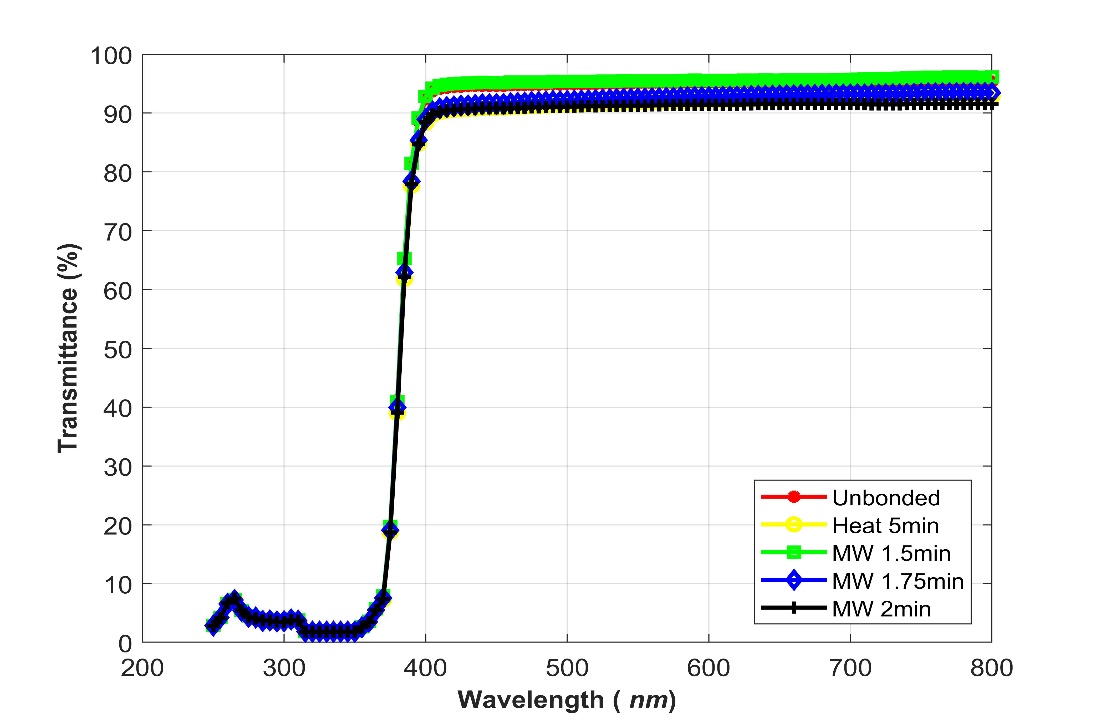
The transmittance (%) evaluated for different configurations of PMMA after bonding is shown in Fig. S2. From the curve, the blank PMMA and 1.5 minutes microwave assisted bonding have nearly equal transmittance. For the other configurations, the transmittance is nearly 3% lesser than the blank PMMA. With more microwave time, the transmittance decreases which is coherent with CA.

**
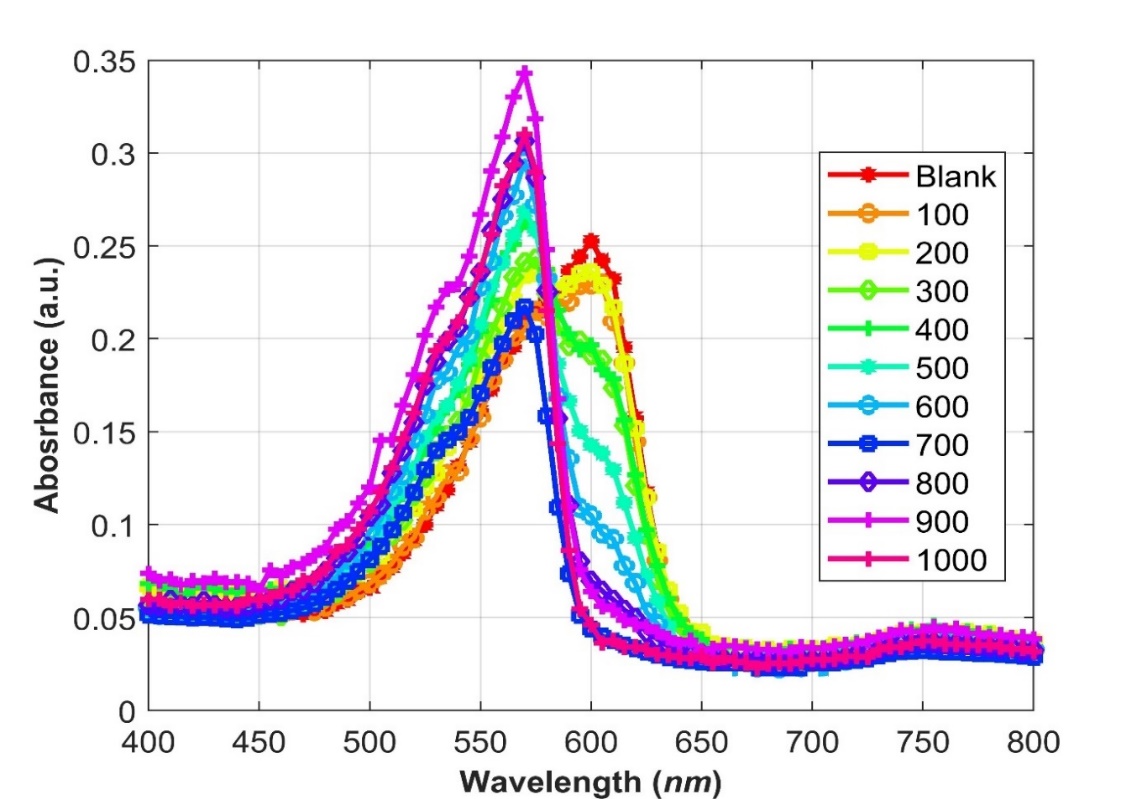
Fig.** **S2.**  The transmittance (%) after bonding for different PMMA configurations. The ‘MW’ represents microwave induced and `Heat' represents heat press based bonding in the figure. Minute is represented by "min" and second by "s".

**Fig.** **S3.** Absorbance spectrum of different concentrations of vitamin C using a spectrophotometer after 10 minutes of addition of resazurin. The legends have the unit µg/mL.


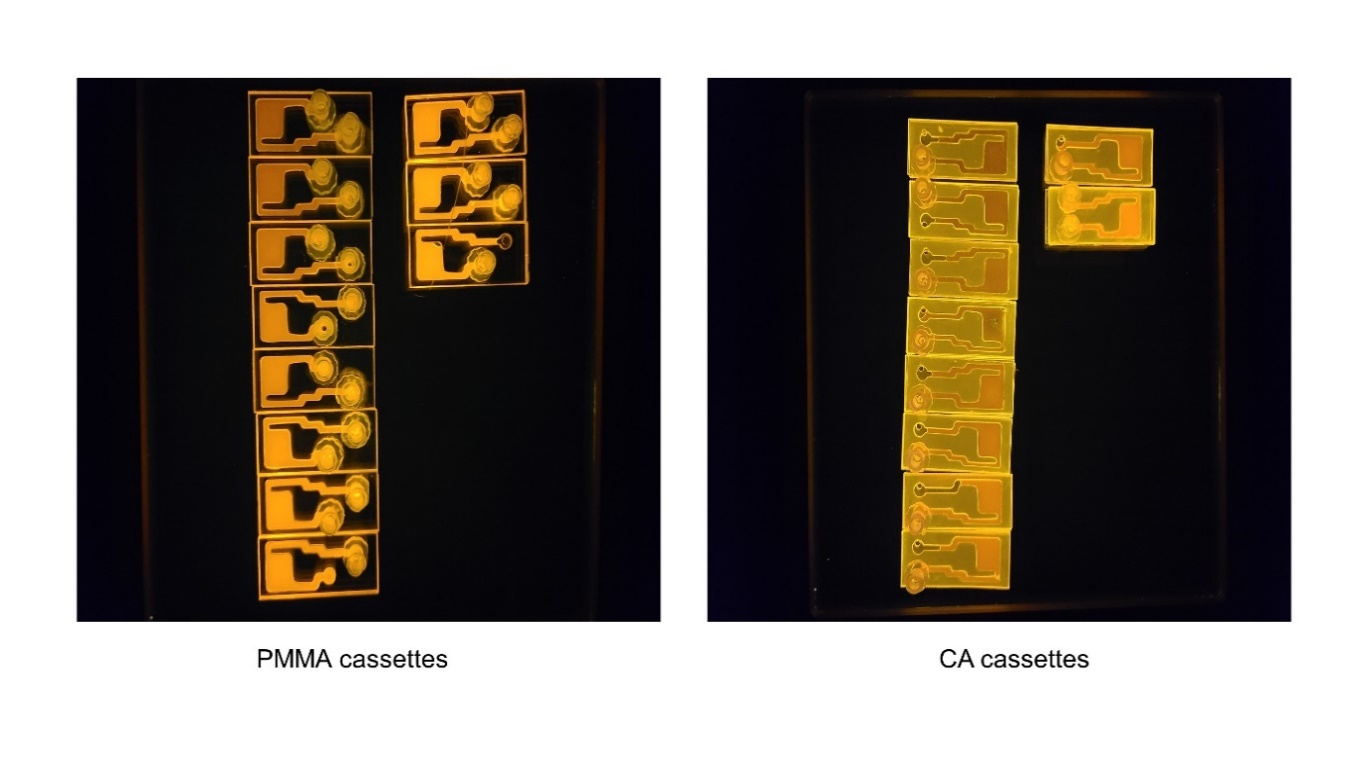
**Fig.** **S4.** The PMMA and CA cassette images for different concentrations inside the safe imager. From top to bottom and then left to right are the cassettes with blank to 1000 µg/mL concentrations of vitamin C after 10 minutes of addition of resazurin. The CA cassettes exhibit autofluorescence.


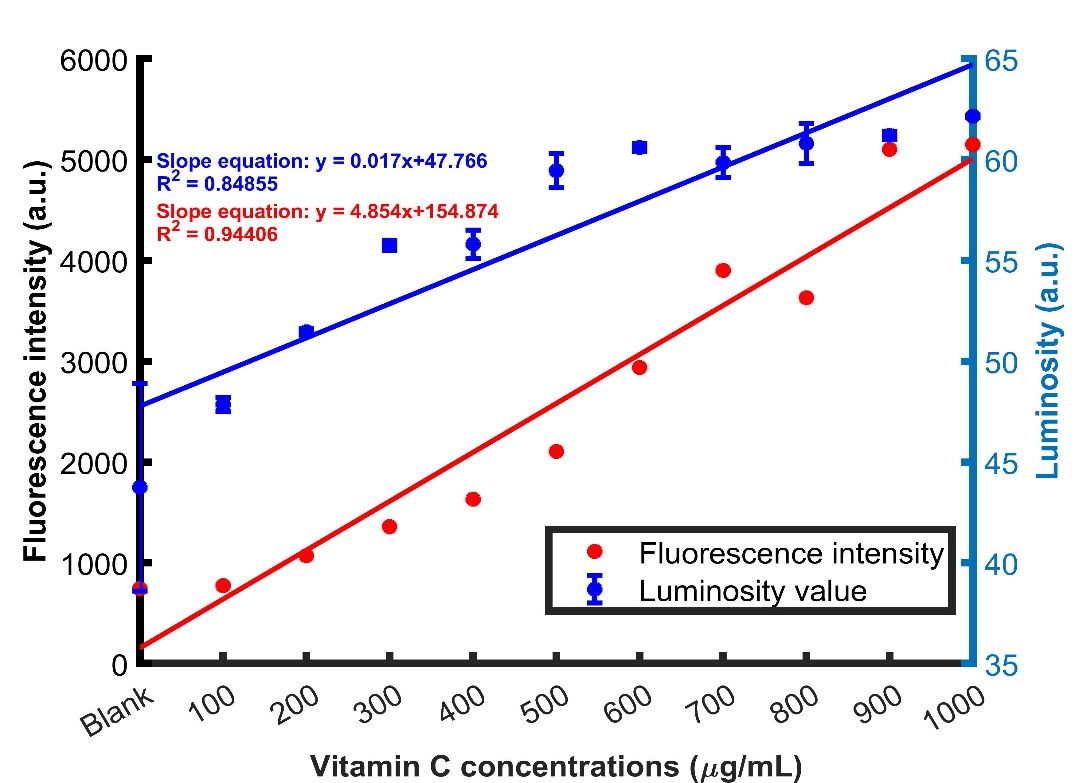
The fluorescence intensities obtained from a spectrophotometer and the luminosity values from the mobile phone-based detector for PMMA is shown in Fig. S5. Although the coefficient of determination (R^2^) of luminosity is lesser than the fluorescence intensity profile, the results from the smartphone camera show a strong correlation with increasing vitamin C concentrations. The linear regression parameters obtained from PMMA and CA cassettes are identical.

**Fig. S5.** Overlap of fluorescence intensities (left y-axis) obtained from a spectrophotometer and the luminosity values (right y-axis) extracted from the mobile phone-based detector for PMMA.

**Table S1.** Leakage test results and burst pressures along with different configurations of PMMA bonding via the heat press and microwave techniques. Two iterations have been performed for each of the tests. Microwave induced bonding strength is higher as similar to the CA. Also, the CA bonding strength is greater than that of PMMA bonding and the time to bond is lesser. This provides dual advantages for the use of the CA sheets over the PMMA bonding.

| Material | Heat press Burst pres-  sure configuration (1*st* itera-  tion) (psi) | Burst pres-  sure  (2*nd* itera- tion) (psi) | Microwave Burst pres-  sure configuration (1*st* itera-  tion) (psi) | Burst pres-  sure  (2*nd* itera- tion) (psi) |
| --- | --- | --- | --- | --- |
| PMMA | 1. minutes Leakage   found   1. minutes Leakage   found | Leakage  found 4 | 1.5 minutes Leakage | 30 |
|  |  |  | found |  |
|  |  |  | 1.75 minutes 28 | 35 |
|  |  |  |  |  |
|  |  |  | 2 minutes 28 | 30 |

**References:**

1. Barril, P., & Nates, S. Introduction to agarose and polyacrylamide gel electrophoresis matrices with respect to their detection sensitivities. *Gel electrophoresis-Principles and basics*, 3-14 (2012).
2. Derua, R., & Bossuyt, X. (2002). Electrophoresis in Practice. A Guide to Methods and Application of DNA and Protein Separations, Reiner Westermeier. *Clinical Chemistry*, **48**(5), 803-804.
3. Witkowska Nery, E., Kundys-Siedlecka, M., Furuya, Y., & Jönsson-Niedziółka, M. Pencil lead as a material for microfluidic 3D-electrode assemblies. *Sensors*, **18**(11), 4037 (2018).
4. Liang, J., Zheng, Y., & Liu, Z. Nanowire-based Cu electrode as electrochemical sensor for detection of nitrate in water. *Sensors and Actuators B: Chemical*, **232**, 336-344 (2016).
5. Gao, W., Song, J., & Wu, N. Voltammetric behavior and square-wave voltammetric determination of trepibutone at a pencil graphite electrode. *Journal of Electroanalytical Chemistry*, **576**(1), 1-7 (2005).
6. Loomatix, https://play.google.com/store/apps/details?id=com.loomatix.
